# Effects of β-alanine supplementation on kickboxing-specific anaerobic performance, neuromuscular power, and strength endurance: A randomized, double-blind, placebo-controlled trial

**DOI:** 10.1371/journal.pone.0346898

**Published:** 2026-05-13

**Authors:** Furkan Hasan Küçük, Cebrail Gençoğlu, Osman Ateş, Jonatas Deivyson Reis Da Silva Duarte, Dino Belošević, Salih Çabuk, Muhammet Raşit İnaç, Süleyman Ulupınar, Serhat Özbay

**Affiliations:** 1 Erzurum City Hospital, Department of Sports Medicine, Erzurum, Türkiye; 2 Erzurum Technical University, Faculty of Sport Sciences, Department of Coaching Education, Erzurum, Türkiye; 3 İstanbul University Cerrahpaşa, Faculty of Sport Sciences, Department of Coaching Education, İstanbul, Türkiye; 4 Graduate Program in Health Sciences, Faculty of Medicine, Federal University of Mato Grosso, Mato Grosso, Brazil; 5 University of Split, Faculty of Kinesiology, Split, Crotia; 6 Erzurum Technical University, Faculty of Sport Sciences, Department of Physical Education and Sports, Erzurum, Türkiye; Dynamical Business & Science Society - DBSS International SAS, COLOMBIA

## Abstract

β-Alanine (BA) elevates skeletal muscle carnosine and may improve repeated high-intensity performance; however, evidence in kickboxing is limited. This study investigated the effects of 4-week BA supplementation on sport-specific kickboxing anaerobic performance, neuromuscular power, and strength endurance in trained male kickboxers. Twenty-eight athletes were randomly assigned to BA (6.4 g·day ⁻ ¹; n = 14) or placebo (n = 14) in a double-blind, placebo-controlled design. Pre- and post-intervention assessments included the Kickboxing Anaerobic Speed Test (KAST₁–₅, KAST_best_, KAST_total_, Performance Decrement Index [PDI]), neuromuscular power (countermovement jump [CMJ], squat jump [SJ]), and strength endurance tests (push-up, pull-up). Baseline differences were tested via *t*-tests, effect sizes calculated according to Hedges’ *g* formula. Group × time effects were analyzed using two-way repeated-measures ANOVA with partial eta squared (ηp²) effect sizes. Baseline measures were comparable (all *p* > 0.05). After supplementation, no significant group × time interactions emerged for CMJ (*p* = 0.148), SJ (*p* = 0.717), KAST₁–₅ (all *p* > 0.05), KAST_best_ (*p* = 0.071), or PDI (*p* = 0.454). However significant improvements were observed in the BA group for KAST_total_ (*F*_(1,26)_ = 14.49, *p* < 0.001, ηp² = 0.358), push-ups (*F*_(1,26)_ = 5.89, *p* = 0.023, ηp² = 0.185), and pull-ups (*F*_(1,26)_ = 9.79, *p* = 0.004, ηp² = 0.274). Four weeks of BA supplementation enhanced total anaerobic performance (KAST_total_) and upper-body strength endurance (push-up, pull-up), while no significant changes were observed in neuromuscular jump performance (CMJ, SJ). BA appears to be a practical ergogenic aid for improving repeated-effort and strength-endurance capacity in kickboxing.

Clinical trial registration

ClinicalTrials.gov (NCT07319052).

## Introduction

β-Alanine is a non-essential amino acid that is naturally present in our daily diet and can also be synthesized by our body in the liver through the catabolism of pyrimidine nucleotides. It holds particular importance for muscle performance as it plays a critical role in carnosine synthesis [1], particularly in type II muscle fibers [2,3]. Carnosine is a dipeptide abundantly stored in the skeletal muscles of vertebrates (including humans) [4]. It is formed by the combination of β-alanine and L-histidine amino acids. Carnosine synthesis is catalyzed by the enzyme carnosine synthase, which requires the presence of both amino acids [1].

The rate-limiting factor for muscle carnosine synthesis is the availability of dietary β-alanine [3]. Supplementation with β-alanine has been shown to increase skeletal muscle carnosine concentrations in both upper and lower extremities [3,5]. Its abundance in skeletal muscle suggests an important role during exercise, with intracellular acid-base regulation being widely accepted as its primary physiological function. However, carnosine has also been proposed to have additional roles including protection against oxidative damage, prevention of glycation, and regulation of calcium sensitivity [6]. These findings have led to investigations into the effects of β-alanine supplementation and consequently elevated muscle carnosine levels on performance and capacity across various exercise protocols and population groups.

Kickboxing on the other hand is a combat sport that involves powerful strikes using the hands, knees, shins, and feet, requiring speed, balance, and strength [7]. The primary objective of kickboxers during a match is to score points and gain superiority by delivering effective kicks and punches to their opponents using proper techniques [8,9]. This sport is often characterized by repeated high-intensity efforts, rapid directional changes, and complex motor tasks, all of which require well-developed neuromuscular coordination, efficient energy metabolism, and a high level of physical endurance [8]. Given the physiological demands imposed by kickboxing, athletes are constantly in pursuit of strategies to optimize performance, accelerate post-training and post-competition recovery, and enhance overall physical resilience [10]. In this context, natural ergogenic aids such as β-alanine have emerged as potential non-doping alternatives for improving athletic performance [5].

The Kickboxing Anaerobic Speed Test (KAST) is a specialized assessment tool designed for kickboxing athletes. The test involves athletes completing a set duration by performing rapid strikes using specific techniques, simulating high-intensity efforts specific to kickboxing [11]. Additionally, it measures the athletes’ anaerobic capacity, explosive speed, and endurance [11]. It has been suggested that β-alanine supplementation can enhance exercise capacity and performance during exercises lasting 0.5–10 minutes [5]. However, there is a lack of specific studies on kickboxing athletes in the existing literature. Complementary to anaerobic performance, explosive lower-limb neuromuscular power is commonly represented by countermovement jump (CMJ) and squat jump (SJ), which serve as valid indicators of stretch–shortening cycle efficiency and rapid force generation in combat sports [12]. In parallel, upper-body strength endurance is frequently characterized by functional calisthenic tasks such as push-ups and pull-ups, which reflect repeated force production capacity of the shoulder–arm complex [13] and are directly relevant for sustaining striking volume in kickboxing.

Therefore, this study aims to investigate the effects of β-alanine supplementation on anaerobic performance, neuromuscular power, and strength endurance in kickboxing athletes, utilizing the Kickboxing Anaerobic Speed Test (KAST), countermovement jump (CMJ), squat jump (SJ), and upper-body endurance tests (push-up and pull-up), which together provide a comprehensive sport-specific assessment framework. Based on the existing literature, it is hypothesized that β-alanine supplementation may enhance anaerobic performance, improve muscular endurance, and reduce fatigue index in kickboxing athletes.

## Methods

### Ethics and trial registration

The study protocol was conducted in accordance with the ethical principles outlined in the Declaration of Helsinki for research involving human participants. Ethical approval was obtained from the Erzurum Technical University Scientific Research and Publication Ethics Committee (Meeting No: 06, Decision No: 5, Date: 21 April 2025).

Prior to participation, all athletes were fully informed about the purpose, procedures, potential risks, and expected benefits of the study. Written informed consent was obtained from all participants before the initiation of any testing procedures. Participants were also informed of their right to withdraw from the study at any time without any penalty.

The trial was prospectively registered at ClinicalTrials.gov (Identifier: NCT07319052; https://clinicaltrials.gov/study/NCT07319052).

### Study design

This study employed a randomized, double-blind, placebo-controlled, parallel-group experimental design to investigate the effects of four weeks of β-alanine supplementation on kickboxing-specific anaerobic performance and neuromuscular endurance in trained male kickboxers.

A total of twenty-eight athletes were randomly allocated into two groups: a β-alanine supplementation group (BA, n = 14) and a placebo control group (CON, n = 14). The intervention period lasted four weeks, during which participants maintained their regular kickboxing training routines under the supervision of their coaches.

All outcome assessments were conducted at two time points: baseline (pre-supplementation) and following the four-week intervention period (post-supplementation). Testing sessions were carried out under standardized laboratory conditions, with ambient temperature maintained between 21–23 °C and relative humidity between 45–55%.

To minimize circadian influences on performance outcomes, all measurements were performed at approximately the same time of day for each participant (±1 hour). Participants were instructed to maintain their habitual dietary patterns and regular kickboxing training routines throughout the intervention period. They were also asked not to introduce any additional training modalities, nutritional supplements, or ergogenic aids during the study.

Participant recruitment commenced on April 30, 2025, primary outcome data collection was completed on May 30, 2025, and the study was finalized on June 4, 2025.

Kickboxing specific anaerobic performance was assessed using the Kickboxing Anaerobic Speed Test (KAST), which included five consecutive sets (KAST₁–₅), the best performance time (KAST_best_), the total time (KAST_total_), and the performance decrement index (PDI). In addition, neuromuscular power and muscular endurance were evaluated using countermovement jump (CMJ), squat jump (SJ), push-up, and pull-up tests.

### Participants

A total of twenty-eight male kickboxers voluntarily participated in this study. The athletes were classified in a combined manner as Highly Trained/National Level and Elite/International Level according to previously established classification criteria [14]. The athletes had a mean training age of 6.64 ± 2.21 years in the BA and 6.57 ± 2.24 years in the CON. In addition, the competitive experience of the athletes was recorded separately. The BA had a mean competitive experience of 4.36 ± 1.28 years, whereas the CON had a mean competitive experience of 4.21 ± 1.34 years. Prior to participating in official competitions, athletes typically completed approximately two years of structured technical training.

Inclusion criteria required that participants:

- had a minimum of five years of systematic kickboxing training experience,
- were actively engaged in regular training and competition within the previous year,
- had no history of musculoskeletal injuries or chronic medical conditions that could interfere with high-intensity exercise performance, and
- were not using any ergogenic aids, nutritional supplements, or medications known to influence physical performance during the study period.

**Fig 1:**
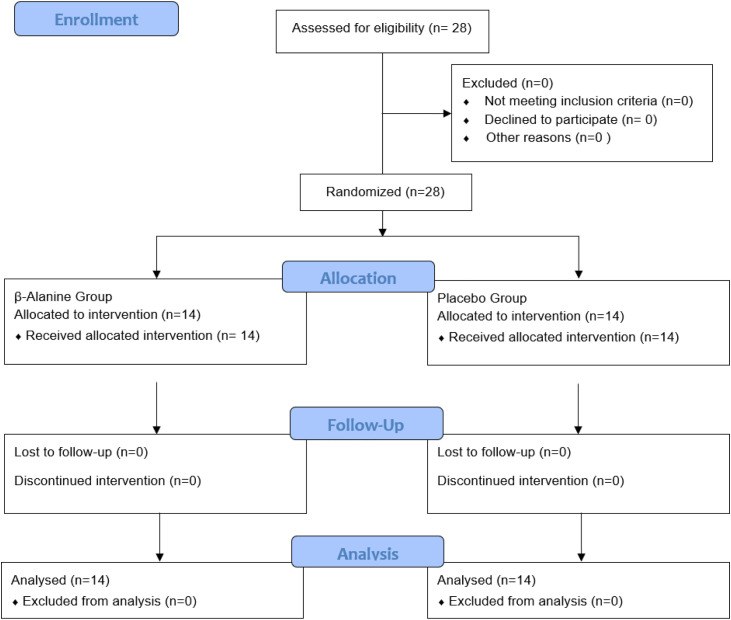
CONSORT flow diagram of participant enrollment, allocation, follow-up, and analysis.

Participant flow throughout the study is presented in Fig 1.

### Sample size calculation

The required sample size was determined a priori using G*Power (version 3.1.9.4) based on a repeated-measures ANOVA model with a within–between interaction. Parameters were set as follows: effect size f = 0.30, α error probability = 0.05, power (1–β) = 0.80, number of groups = 2, number of measurements = 2, correlation among repeated measures = 0.5, and nonsphericity correction ε = 1. The selected effect size corresponds to a moderate magnitude according to established conventions for ANOVA analyses [15] and is consistent with effect magnitudes reported in previous supplementation studies examining performance outcomes [16]. This analysis indicated that a minimum total sample size of 24 participants would be required to detect the expected interaction effect with adequate statistical power. The final sample of 28 athletes therefore exceeded this requirement and provided an actual statistical power of 0.802.

### Supplementation protocol

**Fig 2:**
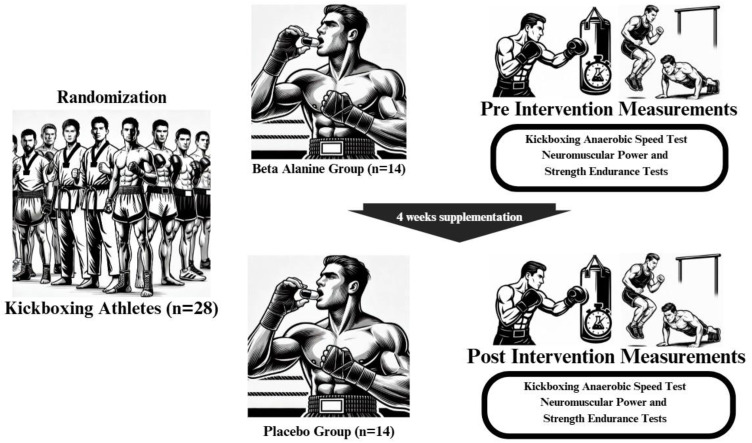
Graphical representation of the supplementation protocol and study design.

Participants in the β-alanine (BA) group consumed 6.4 grams of BA daily for a duration of 4 weeks, in line with previous research protocols [17]. The BA supplement used in this study was Hardline β-alanine (Hardline Nutrition, Türkiye), a commercially available formulation standardized for active ingredient content as illustrated in Fig 2. To ensure methodological rigor and minimize potential expectancy effects, participants in the control group received identical-looking scoops filled with rice flour [17]. All participants were instructed to maintain their habitual exercise and dietary routines throughout the study period. Additionally, they were asked to refrain from using other nutritional supplements, ergogenic aids, and caffeine-containing products.

### Randomization

Participants were randomly allocated to either the β-alanine supplementation group (BA) or the placebo control group (CON) using a stratified randomization procedure to ensure an equal distribution of athletes according to competitive level. Prior to allocation, participants were stratified based on their performance classification (Highly Trained/National Level and Elite/International Level). Within each stratum, athletes were assigned to one of the two experimental conditions using a computer-generated randomization sequence created with an online randomization tool (https://www.randomizer.org/).

The randomization sequence was generated by a researcher who was not involved in the data collection or performance testing procedures. Allocation resulted in 14 athletes in the β-alanine group and 14 athletes in the placebo group, ensuring balanced group sizes and comparable baseline characteristics between groups.

### Blinding

The study followed a double-blind design, whereby both participants and investigators responsible for outcome assessments were blinded to group allocation throughout the intervention period. The β-alanine supplement and placebo were prepared in identical containers and administered using identical scoops, ensuring that both conditions were indistinguishable in appearance and administration procedure.

To maintain allocation concealment, the randomization codes were held by a researcher who was not involved in the testing sessions, participant supervision, or statistical analyses. The allocation codes were revealed only after the completion of all data collection and statistical analyses.

### Outcome measures

#### Anthropometric measurements.

Anthropometric characteristics of the participants were assessed prior to the performance testing sessions. Body height was measured using a wall-mounted stadiometer (Seca, Hamburg, Germany) to the nearest 0.1 cm while participants stood barefoot in an upright position with the head aligned in the Frankfort horizontal plane.

Body mass, body fat percentage, and fat-free mass were determined using a bioelectrical impedance analysis device (Tanita BC-418 MA, Tanita Corp., Tokyo, Japan). Measurements were performed with participants wearing light clothing and barefoot, according to the manufacturer’s guidelines. Prior to the assessment, participants were instructed to avoid strenuous physical activity and food intake for at least 3 hours to minimize potential variability in bioelectrical impedance readings.

Body mass index (BMI) was calculated using the standard equation:


$$BMI=bodymass(kg)/{height}^{\text{2}}\left(m^{\text{2}}\right)$$

All anthropometric assessments were conducted under standardized laboratory conditions and were performed by the same investigator to ensure measurement consistency.

#### Kickboxing Anaerobic Speed Test (KAST) Protocol.

The Kickboxing Anaerobic Speed Test (KAST) was employed as a sport-specific assessment tool to replicate the intermittent anaerobic requirements of competitive kickboxing. In this protocol, athletes were instructed to execute a predetermined four-technique striking sequence at maximal intensity, repeated consecutively for five sets [11]. The standardized sequence consisted of a left jab, right roundhouse kick directed to the trunk, right cross, and left roundhouse kick to the trunk, yielding a total of 20 striking actions per set (10 punches and 10 kicks). For southpaw athletes, the combination was mirrored to maintain stance specificity (right jab, left roundhouse kick, left cross, right roundhouse kick). These movement patterns were chosen due to their frequent application in competitive bouts and their validation in prior scientific investigations [18]. A demonstration of the test application procedure is available in a video format (Scientific Kickboxing Test; Kickboxing Anaerobic Speed Test – Application Procedures: https://www.youtube.com/watch?v=rI628P3XtVQ&t=199s).

Prior to the commencement of testing, participants completed a structured warm-up including five minutes of light running, dynamic bodyweight drills, plyometric exercises, mobility-focused stretching, and shadowboxing routines, as recommended in earlier protocols [11]. A familiarization session was also conducted on a separate day to ensure technical consistency and reduce potential learning effects. During testing, athletes received standardized verbal encouragement to ensure maximal effort output. All procedures were overseen by the same licensed coach, an internationally experienced kickboxing practitioner, to ensure methodological consistency and technical validity.

**Fig 3:**
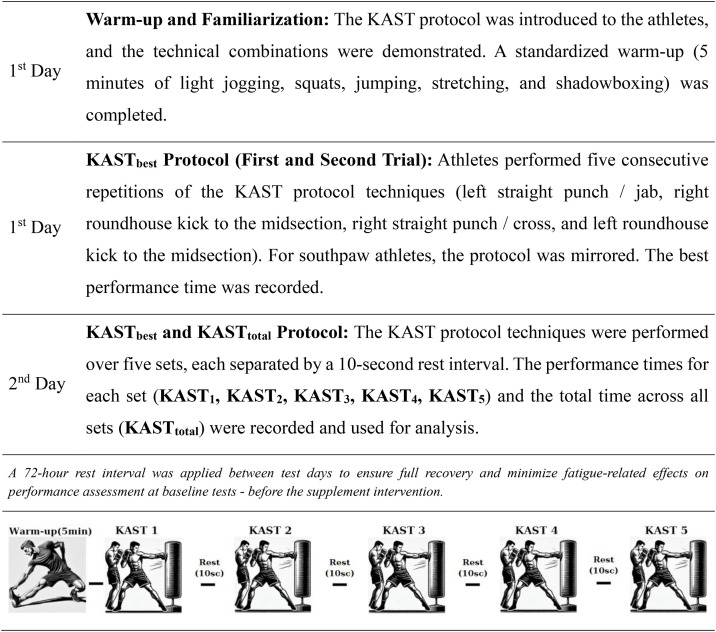
Implementation flowchart of the kickboxing anaerobic speed test (KAST).

For the determination of **KAST**_**best**_, athletes performed two maximal-effort sets of the designated striking sequence, separated by a five-minute passive recovery interval. The faster of the two trials was recorded as the individual’s best score (KAST_best_) for subsequent analyses. To assess cumulative anaerobic performance, the **KAST**_**total**_ protocol required athletes to perform five consecutive sets of the same combination, each separated by 10 seconds of passive recovery. Individual set times (KAST₁–KAST₅) were recorded, and the total duration across all five sets was used as the KAST_total_ measure, reflecting anaerobic performance capacity under repeated high-intensity efforts as summarized in Fig 3.

To quantify fatigue across sets, the **Performance Decrement Index (PDI)** was calculated. This parameter expresses the decline in output relative to the best performance and was computed using the following equation:


$$PDI(\%)=\frac{𝐊𝐀𝐒𝐓𝐭𝐨𝐭𝐚𝐥}{𝐊𝐀𝐒𝐓𝐛𝐞𝐬𝐭x5\left(numberofsets\right)}x100$$

PDI=PerformanceDecrementIndex(%)

where higher values indicate a greater degree of performance decrement across repeated bouts.

All trials commenced following a standardized auditory countdown (“3, 2, 1, ready”), with timing initiated at the moment of first contact with the heavy bag and concluded upon completion of the final kick. Any deviation from the prescribed sequence led to immediate termination of the trial, which was then repeated after a mandatory 10-minute rest interval. Performance times were video-recorded at 25 frames per second and later analyzed using Adobe Premiere Pro (Adobe Inc., USA) to ensure precise time measurement. To control for fatigue accumulation and circadian influences, all testing sessions were scheduled at the same time of day, and at least 72 hours of recovery was provided between repeated test days [11,19].

#### Neuromuscular power tests.

Lower limb neuromuscular power was assessed using the countermovement jump (CMJ) and squat jump (SJ) tests, analyzed with the MyJump mobile application (Apple Store, MyJump2, Spain) [20]. After a standardized warm-up, participants positioned themselves upright with feet shoulder-width apart and toes pointing forward.

For the CMJ, participants started from a standing posture with hands fixed on the hips to exclude arm contribution. They were instructed to perform a rapid knee flexion (~90°) followed immediately by a maximal vertical jump during the concentric phase [21]. For the SJ, participants also placed their hands on the hips, flexed their knees to approximately 90°, and held this position for three seconds before executing an explosive jump to maximum height. The MyJump application, validated for jump height assessment, was used to record and analyze flight time and jump performance.

#### Strength endurance tests.

Upper-body strength endurance was determined by push-up and pull-up tests [7,13] All assessments were conducted on martial arts tatami mats, following self-selected warm-up routines involving dynamic stretching and calisthenics.

Push-ups were executed from a front leaning rest position, with hands placed approximately shoulder-width apart and feet up to 0.3 m apart. A rigid body alignment from ankles to shoulders was maintained throughout. Each repetition required full elbow extension at the top and lowering the chest close to the ground without contact. The maximum number of uninterrupted, correctly executed repetitions was taken as the score [21].

Pull-up performance was assessed using a horizontal bar, with a pronated grip slightly wider than shoulder-width [22]. Participants moved from full arm extension to elbow flexion until the chin cleared the bar. Swinging or compensatory movements were strictly prohibited, and only full-range repetitions were counted.

### Statistics

All analyses were performed using IBM SPSS Statistics for Windows, Version 26.0 (IBM Corp., Armonk, NY, USA). Descriptive data are reported as mean ± standard deviation (SD). Prior to inferential testing, assumptions were examined as follows: normality via Shapiro–Wilk tests and visual inspection of Q–Q plots, between-group homogeneity of variance via Levene’s test, and sphericity for repeated-measures models via Mauchly’s test. The Shapiro–Wilk tests indicated that all variables were normally distributed (p > 0.05). Homogeneity of variance assumptions were satisfied according to Levene’s test (p > 0.05). Mauchly’s test indicated that the assumption of sphericity was not violated; therefore, no Greenhouse–Geisser corrections were required. Potential outliers were screened using boxplots and standardized z-scores (|z| > 3.0), and no cases met the exclusion criteria.

Baseline differences between the β-alanine (BA) and control (CON) groups were assessed using independent-samples t-tests for all variables, including anthropometric characteristics, Kickboxing Anaerobic Speed Test (KAST) outcomes, countermovement jump (CMJ), squat jump (SJ), push-up, and pull-up performance.Test statistics are presented as *t*_(*df*)_ with two-tailed significance levels. Training-independent effects of the four-week supplementation were evaluated with two-way repeated-measures ANOVA models with factors group (BA vs. CON) and time (pre vs. post). Separate models were run for each outcome: KAST_1–5_, KAST_best_, KAST_total_, Performance Decrement Index (PDI), CMJ, SJ, push-up, and pull-up. The primary term of interest was the group × time interaction; statistics are reported as *F*_(1,26)_, *p*, and partial eta-squared ($\overset{\text{2}}{\underset{P}{\eta }}$).Where a significant interaction was detected, simple-effects analyses (Bonferroni-adjusted paired or independent *t* t-tests, as appropriate) were conducted to locate pre-to-post changes within each group and between-group differences at each time point. Effect sizes were also calculated for the practical interpretation of results, allowing the magnitude of observed differences to be evaluated beyond statistical significance. For baseline *t* t-tests, Hedges’ *g* (small-sample corrected) was calculated using the pooled SD [23]. Magnitudes were interpreted using Hopkins’ scale: trivial (|g| < 0.20), small (0.20–0.59), moderate (0.60–1.19), large (≥ 1.20) and very large (≥ 4.0) [24]. For ANOVA terms, $\overset{\text{2}}{\underset{P}{\eta }}$ was interpreted using conventional thresholds: small (≈ 0.01), medium (≈ 0.06), and large (≈ 0.14). In tables, $\overset{\text{2}}{\underset{P}{\eta }}$ values are accompanied by magnitude labels in subscript (e.g., 0.079 Medium Effect; _ME_). The alpha level was set at *p* = 0.05. All *t* and *F* degrees of freedom are forma*t*ted as subscripts [e.g., *t*_(26)_, *F*_(1,26)_] *t*o match the reporting style in Tables 1–5.

**Table 1:** Anthropometric characteristics and train experience of the groups.

	Beta Alanine Group	Control Group	*t*_(26)_	P	ES (Hedge’s g)
**Age**_**(year)**_	24.29 ± 3.79	26.21 ± 4.51	−1.225	0.232	−0.45_SES_
**Body Mass**_**(kg)**_	67.94 ± 7.67	69.35 ± 7.31	−0.497	0.623	−0.18_TES_
**Height**_**(cm)**_	174.73 ± 7.60	174.10 ± 8.81	0.205	0.839	0.07_TES_
**BMI**_**(kg/height [m²])**_	22.21 ± 1.61	22.90 ± 2.09	−0.972	0.340	−0.35_SES_
**Body Fat**_**(%)**_	8.71 ± 2.22	9.92 ± 3.06	−1.196	0.242	−0.44_SES_
**Fat Free Mass**_**(kg)**_	61.75 ± 6.28	62.39 ± 6.10	−0.273	0.787	−0.10_TES_
**Train Age**_**(year)**_	6.64 ± 2.21	6.57 ± 2.24	0.085	0.933	0.03_TES_

Note: Data are presented as mean ± standard deviation; BMI: Body mass index; ES: Effect size (Hedge’s g formula); SES: Small effect size; TES: Trivial effect size.

**Table 2:** Kickboxing anaerobic speed test performances comparison before starting the supplement intervention protocol.

	Beta Alanine Group	Control Group	*t*_(26)_	P	ES (Hedge’s g)
**KAST**_**1**_	7.34 ± 0.55	7.49 ± 0.52	−0.776	0.445	−0.28_SES_
**KAST**_**2**_	7.36 ± 0.57	7.51 ± 0.60	−0.723	0.476	−0.27_SES_
**KAST**_**3**_	7.88 ± 0.67	8.09 ± 0.70	−0.795	0.434	−0.29_SES_
**KAST**_**4**_	8.31 ± 0.91	8.89 ± 1.11	−1.522	0.140	−0.56_SES_
**KAST**_**5**_	9.46 ± 1.02	10.08 ± 1.44	−1.319	0.199	−0.48_SES_
**KAST**_**best**_	7.05 ± 0.49	7.20 ± 0.39	−0.879	0.388	−0.33_SES_
**KAST**_**total**_	40.36 ± 2.34	42.08 ± 3.18	−1.634	0.114	−0.60_MES_
**PDI**_**(%)**_	14.98 ± 9.68	17.05 ± 7.15	−0.645	0.525	−0.23_SES_

Note: Data are presented as mean ± standard deviation; KAST 1–5: Kickboxing Anaerobic Speed Test scores round 1^st^ to 5^th^; KAST_best_: Best KAST score; KAST_total_: Total KAST score; PDI: Performance Decrement Index (%); ES: Effect size (Hedge’s g formula); SES: Small effect size; TES: Trivial effect size, MES: Moderate effect size.

**Table 3:** Baseline comparisons of physical performance before starting the supplement intervention7protocol.

	Beta Alanine Group	Control Group	*t*_(26)_	P	ES (Hedge’s g)
**CMJ (cm)**	34.20 ± 4.83	33.21 ± 5.75	0.493	0.626	0.18_TES_
**SJ (cm)**	25.39 ± 4.02	26.30 ± 5.20	−0.519	0.608	−0.19_TES_
**Push-up (n)**	54.86 ± 15.00	50.86 ± 9.89	0.833	0.412	0.31_SES_
**Pull-up (n)**	19.79 ± 5.49	18.57 ± 6.73	0.523	0.606	0.19_TES_

Note: Data are presented as mean ± standard deviation; CMJ = Countermovement Jump; SJ = Squat Jump; ES = effect size (Hedge’s g, with Hopkins’s classification); ES: Effect size (Hedge’s g formula); SES: Small effect size; TES: Trivial effect size.

**Table 4:** Kickboxing anaerobic speed test performances, group*time intervention after Beta Alanine supplementation.

	Beta Alanine Group		Control Group		Group*Time ES ($\overset{2}{\underset{P}{\eta }}\overset{2}{\underset{P}{\eta }}$)
	Before	After	Before	After	
**KAST**_**1**_	7.34 ± 0.55	7.04 ± 0.57	7.49 ± 0.52	7.38 ± 0.58	0.097_ME_
**KAST**_**2**_	7.36 ± 0.57	7.10 ± 0.54	7.52 ± 0.60	7.47 ± 0.55	0.061_ME_
**KAST**_**3**_	7.88 ± 0.67	7.52 ± 0.69	8.09 ± 0.70	7.92 ± 0.66	0.083_ME_
**KAST**_**4**_	8.31 ± 0.91	8.07 ± 1.03	8.90 ± 1.11	8.76 ± 1.18	0.026_SE_
**KAST**_**5**_	9.46 ± 1.02	9.13 ± 1.04	10.08 ± 1.44	9.96 ± 1.42	0.134_ME_
**KAST**_**best**_	7.05 ± 0.49	6.75 ± 0.50	7.20 ± 0.39	7.14 ± 0.47	0.120_ME_
**KAST**_**total**_	40.36 ± 2.34	38.85 ± 2.46	42.09 ± 3.18	41.50 ± 3.63	**0.358**_**LE**_ **ǂ**
**PDI**_**(%)**_	14.98 ± 9.68	15.52 ± 10.16	17.05 ± 7.15	16.22 ± 6.01	0.022_SE_

Data are presented as mean ± standard deviation; level. **ǂ**: Indicates a significant group × time interaction at p < 0.05 level; KAST₁–₅: Kickboxing Anaerobic Speed Test scores from 1^st^ to 5^th^ sets; KAST_best_: Best KAST score; KAST_total_: Total KAST score; PDI: Performance Decrement Index (%); ES: Effect size;$\overset{\text{2}}{\underset{P}{\eta }}\overset{\text{2}}{\underset{P}{\eta }}$: Partial eta squared; ME: Medium effect in group*time interaction; LE: Large effect in group*time interaction.

**Table 5:** Neuromuscular power and strength endurance performances, group*time interaction after Beta Alanine supplementation.

	Beta Alanine Group		Control Group		Group*Time ES ($\overset{2}{\underset{P}{\eta }}\overset{2}{\underset{P}{\eta }}$)
	Before	After	Before	After	
**CMJ (cm)**	34.20 ± 4.83	34.71 ± 4.87	33.21 ± 5.75	33.27 ± 5.68	0.079_ME_
**SJ (cm)**	25.39 ± 4.01	25.52 ± 4.32	26.30 ± 5.20	26.59 ± 5.62	0.005_TE_
**Push-up (n)**	54.86 ± 15.00	59.07 ± 13.08	50.86 ± 9.89	52.36 ± 8.82	**0.185**_**LE**_ **ǂ**
**Pull-up (n)**	19.79 ± 5.49	23.21 ± 6.52	18.57 ± 6.73	19.71 ± 7.04	**0.274**_**LE**_ **ǂ**

Data are presented as mean ± standard deviation. CMJ = Countermovement Jump; SJ = Squat Jump. **ǂ**: Indicates a significant group × time interaction at p < 0.05 level; ES: Effect size;$\overset{\text{2}}{\underset{P}{\eta }}\overset{\text{2}}{\underset{P}{\eta }}$ Partial eta squared; ME: Medium effect in group*time interaction; TE: Trivial effect in group*time interaction; LE: Large effect in group*time interaction.

## Results

No significant differences were observed between the β-alanine and control groups in terms of age, body mass, height, BMI, body fat percentage, fat-free mass, or training age (all *p* > 0.05) as described in Table 1. Specifically, age did not differ between the groups (*t*_(26)_ = −1.23, *p* = 0.232, *g* = −0.45, small effec*t*), and no si*g*nificant difference was detected in body mass (*t*_(26)_ = −0.50, *p* = 0.623, *g* = −0.18, *t*rivial effect). Similarly, no between-*g*roup differences were found for height (*t*_(26)_ = 0.21, *p* = 0.839, *g* = 0.07, *t*rivial effect), BMI (*t*_(30)_ = −0.97, *p* = 0.340, *g* = −0.35, small effec*t*), or body fat percenta*g*e (*t*_(26)_ = −1.20, *p* = 0.242, *g* = −0.44, small effec*t*). Fat-free mass was comparable across *g*roups (*t*_(26)_ = −0.27, *p* = 0.787, *g* = −0.10, *t*rivial effect), and trainin*g* age also showed no significant differences (*t*_(26)_ = 0.09, *p* = 0.933, *g* = 0.03, *t*rivial effect).

Table 2 revealed no significant between-group differences in KAST performances prior to the supplementation protocol (all p > 0.05). Specifically, KAST₁ times were similar between the β-alanine and control groups (t_(26)_ = −0.78, p = 0.445, g = −0.29, small effect), as were KAST₂ (t_(26)_ = −0.72, p = 0.476, g = −0.27, small effect) and KAST₃ (t_(26)_ = −0.80, p = 0.434, g = −0.29, small effect). Likewise, KAST₄ (t_(26)_ = −1.52, p = 0.140, g = −0.56, small-to-moderate effect) and KAST₅ (t_(26)_ = −1.32, p = 0.199, g = −0.48, small effect) did not differ significantly between groups. Moreover, the best performance (KAST_best_) was not statistically different (t_(26)_ = −0.88, p = 0.388, g = −0.33, small effect). Total performance time (KAST_total_) also showed no significant group difference (t_(26)_ = −1.63, p = 0.114, g = −0.60, moderate effect), and no differences were observed in PDI (t_(26)_ = −0.65, p = 0.525, g = −0.24, trivial effect).

As shown in Table 3, no significant between-group differences were found in baseline physical performance variables (all p > 0.05). Specifically, CMJ performance was comparable between the Beta Alanine and Control groups (t_(26)_ = 0.49, p = 0.626, g = 0.18, trivial effect size), as was SJ performance (t_(26)_ = −0.52, p = 0.608, g = −0.19, trivial effect size). Similarly, no significant differences were observed in upper-body muscular endurance, with push-up performance showing small, non-significant differences (t_(26)_ = 0.83, p = 0.412, g = 0.31, small effect size), and pull-up performance also remaining comparable across groups (t_(26)_ = 0.52, p = 0.606, g = 0.19, trivial effect size).

**Fig 4:**
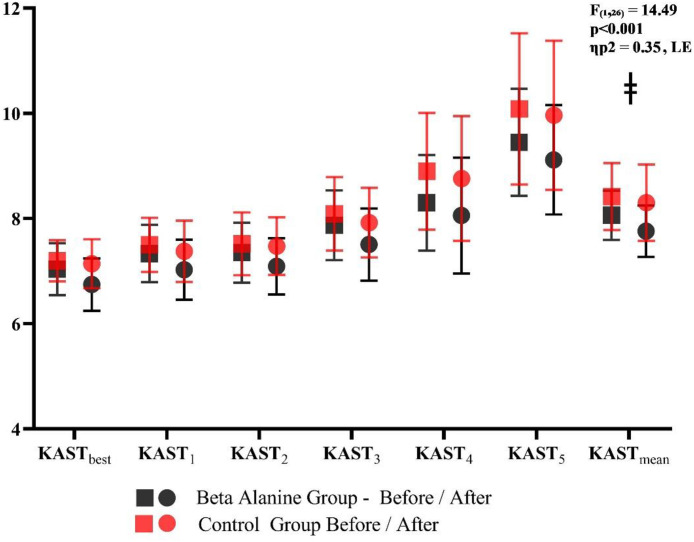
Kickboxing anaerobic speed test performance scores before and after supplementation protocol. Notes; ǂ: Significant group*time interaction (p < 0.05); KAST_mean_: KAST_total_/5.

The two-way repeated measures ANOVA revealed no statistically significant group × time interactions for KAST₁ (F_(1,26)_ = 2.80, p = 0.106, $\overset{\text{2}}{\underset{P}{\eta }}$ = 0.097), KAST₂ (F_(1,26)_ = 1.69, p = 0.205, $\overset{\text{2}}{\underset{P}{\eta }}$= 0.061), KAST₃ (F_(1,26)_ = 2.34, p = 0.138, $\overset{\text{2}}{\underset{P}{\eta }}$ = 0.083), KAST₄ (F_(1,26)_ = 0.68, p = 0.417, $\overset{\text{2}}{\underset{P}{\eta }}$ = 0.026), or KAST₅ (F_(1,26)_ = 4.03, p = 0.055, $\overset{\text{2}}{\underset{P}{\eta }}$ = 0.134). Similarly, the group × time effect did not reach statistical significance for KAST_best_ (F_(1,26)_ = 3.55, p = 0.071, $\overset{\text{2}}{\underset{P}{\eta }}$ = 0.120) or for the performance decrement index (PDI) (F_(1,26)_ = 0.58, p = 0.454, $\overset{\text{2}}{\underset{P}{\eta }}$ = 0.022). However, a significant interaction effect was observed for KAST_total_ (F_(1,26)_ = 14.49, p < 0.001, $\overset{\text{2}}{\underset{P}{\eta }}$ = 0.358), indicating a differential adaptation between groups across the supplementation period (Table 4 and Fig 4).

As presented in Table 5 and Fig 4, the two-way repeated measures ANOVA revealed no significant group × time interaction for countermovement jump (CMJ) performance (*F*_(1,26)_ = 2.23, *p* = 0.148, $\overset{\text{2}}{\underset{P}{\eta }}$ = 0.079). Similarly, squat jump (SJ) performance did not show a significant group × time interaction (*F*_(1,26)_ = 0.13, *p* = 0.717, $\overset{\text{2}}{\underset{P}{\eta }}$ = 0.005). In contrast, significant group × time interactions were detected in the upper-body performance tests. Push-up repetitions demonstrated a significant interaction effect between the Beta Alanine and Control groups across time (*F*_(1,26)_ = 5.89, *p* = 0.023, $\overset{\text{2}}{\underset{P}{\eta }}$ = 0.185). Likewise, pull-up repetitions revealed a significant group × time interaction (*F*_(1,26)_ = 9.79, *p* = 0.004, $\overset{\text{2}}{\underset{P}{\eta }}$ = 0.274). Taken together, the findings in Table 5 indicate that while lower-body explosive performance measures (CMJ and SJ) remained unaffected, the upper-body performance measures (push-up and pull-up) exhibited statistically significant group × time interaction effects.

## Discussion

This study holds particular importance within the field of sports sciences as it represents, to our knowledge, this is the first study to evaluate the effects of β-alanine supplementation using a sport-specific performance test tailored for kickboxing athletes. While most previous research has relied on general laboratory-based protocols, the implementation of the Kickboxing Anaerobic Speed Test (KAST) provided a unique opportunity to assess anaerobic capacity and fatigue resistance in a setting closely reflecting the physiological and technical demands of competitive bouts. The findings revealed meaningful group × time interaction effects, particularly for KASTtotal, where the β-alanine group improved from 40.36 ± 2.34 s at baseline to 38.85 ± 2.46 s after supplementation, while the control group showed only a minimal change (42.09 ± 3.18 s to 41.50 ± 3.63 s). Similarly, upper-body strength endurance measures demonstrated clear improvements in the β-alanine group, with push-up repetitions increasing from 54.86 ± 15.00 to 59.07 ± 13.08 and pull-ups from 19.79 ± 5.49 to 23.21 ± 6.52, whereas the control group exhibited only negligible changes across the intervention period. These results emphasize that β-alanine supplementation can yield performance benefits specifically aligned with the demands of combat sports, thereby supporting its practical application in training and competition contexts.

Specifically in combat sports, previous studies have reported performance improvements resulting from β-alanine supplementation in sport-specific tests for Boxing [25] and judo [26]. However, studies employing non–sport-specific tests have also demonstrated ergogenic effects, such as increased total work output in the Wingate test among jiu-jitsu [27], boxing [28] and judo athletes [29], as well as a reduction in mean sprint time over 300 yards in wrestlers [30]. These findings are consistent with the results of the present study. Moreover, four weeks of strength training combined with β-alanine supplementation reduced the fatigue index in the Wingate test among boxers [31].

In our case, we employed a sport-specific test (KAST), whose main advantage lies in its ability to more accurately replicate the technical and physiological demands of real combat situations, thereby providing measures with greater ecological validity and practical applicability compared to traditional laboratory-based assessments [32]. Nevertheless, the lack of additional studies examining the effects of β-alanine supplementation using sport-specific tests in different combat sports limits the possibility of broader direct comparisons.

Other studies have also demonstrated ergogenic effects of β-alanine supplementation in various sports, such as improved sprint performance in rugby, wrestling, and water polo athletes [33–35] as well as reduced completion times in 100- and 200-meter swimming events [36] and 2,000-meter rowing [37]. However, investigations examining 20 km cycling performance [38], 5,000-meter running time [39], sets of 5 sprints of 5 seconds each with 45 seconds of recovery, separated by 2 minutes of active recovery [40] and CMJ performance in basketball players [41] did not demonstrate ergogenic effects of β-alanine—findings that align with our results for CMJ and SJ.

A possible explanation for the absence of ergogenic effects in certain tests and sports is that the duration of the exercise may be either too short or too long to sufficiently stress the anaerobic glycolytic metabolism and, consequently, induce a meaningful accumulation of hydrogen ions (H⁺) [26]. Supporting this notion, a recent meta-analysis demonstrated that β-alanine supplementation is most effective in enhancing high-intensity exercise performance lasting between 4 and 10 minutes [42].

From a physiological standpoint, a possible explanation for the performance improvements observed in the KAST, push-up, and pull-up tests is related to enhanced anaerobic resistance. Short-duration, high-intensity exercises lead to a pronounced activation of the glycolytic pathway, resulting in the accumulation of intracellular H⁺ [43]. The increased H⁺ concentration in skeletal muscle competes with calcium ions for binding sites on troponin, thereby impairing the formation of actin–myosin cross-bridges [44]. Moreover, muscular acidosis can compromise mitochondrial function and enzymatic activity (phosphocreatine), as well as induce membrane hyperpolarization, which reduces the rate of nerve impulse propagation [45]. Collectively, these mechanisms contribute to marked muscular fatigue and hinder the maintenance of high-intensity actions over prolonged periods [46]. Conversely, the increase in intracellular carnosine induced by β-alanine supplementation acts as the primary intracellular H⁺ buffer, allowing athletes to better tolerate efforts that demand high glycolytic activation [1,47].

Specifically in kickboxing, β-alanine supplementation appears particularly advantageous for several reasons: (a) matches induce a significant increase in H⁺ concentration [48]; (b) blood lactate levels during bouts can range from 10.23 to 15.8 mmol·L ⁻ ¹ [49–51], reflecting a high demand on anaerobic glycolytic metabolism; and (c) anaerobic energy systems play a determinant role in combat sports [52], with the glycolytic system predominating during prolonged striking exchanges lasting 6–30 seconds. β-Alanine appears to extend the time to exhaustion during high-intensity actions [1,17,53], which is particularly desirable in kickboxing bouts.

Although the present study employed a randomized, double-blind, placebo-controlled clinical trial design — widely recognized as the most robust method for evaluating intervention efficacy due to its ability to minimize selection and confounding biases and provide high-quality evidence [54] — several methodological limitations should be acknowledged. First, muscle biopsies were not performed to directly quantify intramuscular carnosine concentration, which would have allowed confirmation of the physiological effect of β-alanine supplementation on the target tissue. Additionally, physiological markers such as heart rate, blood lactate, and H⁺ concentration were not measured during the tests, variables that could provide a more detailed understanding of exercise intensity, glycolytic pathway activation, and acid-base balance during performance. Finally, some athletes reported episodes of paresthesia during the first week of supplementation, which, although a known side effect of β-alanine [55], may have partially compromised the study’s blinding. This limitation, albeit difficult to control, should be considered when interpreting the results, as subtle subjective perceptions can influence behavioral and motivational responses during performance testing.

### Limitations

Despite the strengths of the present randomized placebo-controlled design, several limitations should be acknowledged. First, intramuscular carnosine concentration was not directly measured through muscle biopsy or non-invasive techniques such as proton magnetic resonance spectroscopy. Therefore, the physiological mechanism underlying the observed performance improvements could not be directly confirmed. Second, physiological markers associated with exercise intensity and metabolic stress, such as blood lactate concentration, hydrogen ion accumulation, or heart rate responses, were not assessed during the performance tests. The inclusion of these variables would provide additional insight into the metabolic demands of the KAST protocol and the potential buffering effects of β-alanine supplementation.

Another limitation relates to the potential influence of paresthesia, a commonly reported side effect of β-alanine supplementation. Some athletes reported transient paresthesia sensations during the initial phase of supplementation. Although the study followed a double-blind design, this sensory effect may have partially influenced participants’ perceptions regarding their group allocation, which could potentially affect the effectiveness of the blinding procedure. Alternative dosing strategies involving smaller divided doses may reduce the prevalence of this sensation. However, the supplementation protocol used in the present study reflects common real-world supplementation practices used by athletes, thereby increasing the ecological validity of the intervention.

In addition, the inclusion of only male kickboxing athletes may limit the generalizability of the findings to other combat sports populations, female athletes, or athletes from different training backgrounds. Future studies should include larger samples, incorporate physiological measurements related to acid–base balance and muscle carnosine levels, and examine longer supplementation periods in order to better understand the mechanisms and broader applicability of β-alanine supplementation in combat sports.

## Conclusion

The present study demonstrated that four weeks of β-alanine supplementation significantly improved total anaerobic performance, as assessed by the Kickboxing Anaerobic Speed Test (KAST_total_), together with upper-body strength endurance (push-up and pull-up) in trained male kickboxers, whereas no meaningful changes were observed in lower-limb explosive power (CMJ, SJ). Although no direct physiological measurements were conducted, the observed improvements may be related to mechanisms previously associated with β-alanine supplementation, such as increased intramuscular carnosine content and enhanced buffering capacity, which may contribute to improved tolerance to repeated high-intensity efforts. In practical terms, β-alanine supplementation appears to be a potentially effective strategy for sustaining striking power and technical performance in kickboxing and similar combat sports requiring repeated explosive actions. Future studies should incorporate physiological markers such as muscle carnosine, lactate kinetics, or muscle oxygenation to clarify the mechanistic basis of these effects and extend investigations to different athlete populations and longer supplementation periods.

## Supporting information

S1 File CONSORT checklist beta alanine. (PDF)

S2 File Study protocol, ethical decision revised. (PDF)
