# Supplementary material for: Effects of β-alanine supplementation on kickboxing-specific anaerobic performance, neuromuscular power, and strength endurance: A randomized, double-blind, placebo-controlled trial
Source: PLoS One. 2026 May 13;21(5):e0346898. doi: 10.1371/journal.pone.0346898 (PMC13170865; doi:10.1371/journal.pone.0346898)
Supplement: S2 File — (PDF) [file pone.0346898.s002.pdf]

STUDY PROTOCOL

(Ethics-Approved Clinical Trial Protocol – Full Version)

Title of the Study: Effects of β-Alanine Supplementation on Kickboxing-Specific Anaerobic Performance, Neuromuscular Power, and Strength Endurance: A Randomized, Double-Blind, Placebo-Controlled Trial

Administrative Information

Institution: Erzurum Technical University, Faculty of Sport Sciences
Ethics Committee: Erzurum Technical University Scientific Research and Publication Ethics Committee
Ethics Approval Date: 21 April 2025
Meeting Number / Decision Number: 06 / 5

Principal Investigator / Corresponding Author:
Assist. Prof. Dr. Cebrail Gençoğlu

Ethics Approval and Regulatory Statement

This study protocol represents the complete and original clinical trial protocol that was submitted to and approved by the Erzurum Technical University Scientific Research and Publication Ethics Committee before participant recruitment and data collection began.

The Ethics Committee **reviewed and approved all aspects of the study**, including:

- study objectives,
- participant eligibility criteria,
- randomization and blinding procedures,
- supplementation protocol,
- testing and measurement procedures,
- data collection and statistical analysis plan,
- informed consent process, and
- participant safety and confidentiality procedures.

The study was conducted in full compliance with the **Declaration of Helsinki** and relevant institutional and national research regulations. No deviations from the approved protocol occurred during the conduct of the trial.

Background and Rationale

β-Alanine supplementation is known to increase intramuscular carnosine concentration, enhancing intracellular buffering capacity during high-intensity exercise. Kickboxing is characterized by repeated high-intensity striking actions that rely heavily on anaerobic glycolytic energy pathways. Despite extensive research on β-alanine in general exercise models, evidence derived from sport-specific kickboxing performance tests remains limited. Therefore, this randomized controlled trial was designed to evaluate the ergogenic effects of β-alanine using validated kickboxing-specific and neuromuscular performance assessments.

Objectives

Primary Objective

To examine the effect of four-week β-alanine supplementation on kickboxing-specific anaerobic performance assessed by the Kickboxing Anaerobic Speed Test (KAST).

Secondary Objectives

To evaluate changes in fatigue indices and total anaerobic performance output.

To assess neuromuscular power using countermovement jump (CMJ) and squat jump (SJ).

To assess upper-body strength endurance using push-up and pull-up tests.

Study Design

This study employed a **randomized, double-blind, placebo-controlled, parallel-group clinical trial design**.

Participants were randomly allocated in a 1:1 ratio to either a β-alanine supplementation group or a placebo group. Both participants and investigators were blinded to group allocation throughout the intervention and testing period.

Participants

Inclusion Criteria

Male kickboxing athletes

Minimum of 5 years of systematic kickboxing training

Actively training and competing

Free from injury and chronic disease

Exclusion Criteria

Presence of chronic medical conditions

Use of nutritional supplements or ergogenic aids within the last 3 months

Failure to comply with supplementation or testing procedures

Written informed consent was obtained from all participants prior to study participation.

Randomization and Blinding

Participants were randomly assigned to groups using a stratified randomization procedure to ensure baseline equivalence. Supplement containers were prepared by an independent researcher not involved in testing or data analysis. Supplements were identical in appearance, taste, and packaging to maintain double blinding.

Supplementation Protocol

Participants in the β-alanine group consumed **6.4 $g \cdot day^{-1}$ of β-alanine** for four weeks. The daily dose was divided into equal portions to minimize potential side effects.

The placebo group received an identical dosage of rice flour. Participants were instructed to maintain their habitual diet and training routines and to refrain from using additional supplements throughout the study.

Outcome Measures

Primary Outcome

Kickboxing Anaerobic Speed Test ($KAST_{1–5}$, KASTbest, KASTtotal, Performance Decrement Index)

Secondary Outcomes

Countermovement Jump (CMJ)

Squat Jump (SJ)

Push-up test

Pull-up test

All assessments were conducted at baseline and after the four-week intervention under standardized laboratory conditions.

Statistical Analysis Plan

Data analysis was pre-specified and approved by the Ethics Committee. Statistical analyses were performed using IBM SPSS Statistics software. Normality assumptions were assessed prior to inferential testing. Group × time effects were analyzed using two-way repeated-measures ANOVA. Statistical significance was set at **$p < 0.05$**.

Data Management and Confidentiality

All participant data were anonymized and stored securely. Only the research team had access to identifiable data. Data were used exclusively for scientific purposes.

Adverse Events and Safety Monitoring

Participants were monitored throughout the study for adverse events. Mild paresthesia associated with β-alanine supplementation was reported by some participants and resolved spontaneously. No serious adverse events occurred.

Ethical Approval

Ethics Committee Approval Statement

The official Ethics Committee approval letter corresponding to this protocol is provided as a supplementary file. Ethical Statement at the manuscript "The study protocol was conducted in accordance with the ethical principles outlined in the Declaration of Helsinki for research involving human participants. Ethical approval was obtained from the Erzurum Technical University Scientific Research and Publication Ethics Committee (Meeting No: 06, Decision No: 5, Date: 21 April 2025)."
